# Ultrasound‐assisted and landmark‐based nusinersen delivery in spinal muscular atrophy adults: A retrospective analysis

**DOI:** 10.1002/ajum.12401

**Published:** 2024-07-15

**Authors:** Bruno Antonio Zanfini, Agata Katia Patanella, Francesco Vassalli, Stefano Catarci, Marika Pane, Luciano Frassanito, Matteo Biancone, Mariangela Di Muro, Chiara Bravetti, Eugenio Maria Mercuri, Mario Sabatelli, Gaetano Draisci

**Affiliations:** ^1^ Department of Emergency, Anesthesiological and Reanimation Sciences Fondazione Policlinico Universitario Agostino Gemelli IRCCS Rome Italy; ^2^ Centro Clinico NeMO Adulti Fondazione Policlinico Universitario Agostino Gemelli IRCCS Rome Italy; ^3^ Obstetric Anesthesia, Department of Critical Care and Perinatal Medicine Istituto di Ricovero e Cura a Carattere Scientifico (IRCCS) Istituto Giannina Gaslini Genoa Italy; ^4^ Centro Clinico NeMO Pediatrico Fondazione Policlinico Universitario A Gemelli IRCCS Rome Italy; ^5^ Università Cattolica del Sacro Cuore Rome Italy

**Keywords:** adult, lumbar spine, nusinersen, spinal muscular atrophy, ultrasound assistance

## Abstract

**Introduction/Purpose:**

Nusinersen, the first treatment approved for all spinal muscular atrophy (SMA) types, is administered intrathecally through lumbar puncture. We used ultrasound assistance or a landmark‐based technique to access the lumbar intrathecal space in adult SMA patients. This study aimed to evaluate the technical success and adverse events (AEs) in such patients using either technique over a long observation period.

**Methods:**

Fifty‐one adult patients received 507 consecutive interlaminar nusinersen administrations. Patients presented with both ‘uncomplicated spines’ or ‘complicated spines’; two patients had previous back surgery. Technical success and AEs were recorded using either technique. A generalised linear mixed model was applied to evaluate predictors of technical success and complications.

**Results:**

An overall success rate of 99.6%, with only two procedures failing to reach the intrathecal space, and an overall optimal procedure rate of 90.3% have been reported. A total of 455 procedures (89.7%) were uneventfully performed. One (0.2%) case of severe AE (puncture of a bulky abdominal annexal cyst) was recorded. Twenty‐seven episodes (5.3%) of post‐dural puncture headache (PDPH) and 24 episodes (4.7%) of radicular or back pain, both successfully treated with medical therapy, have also been reported. Technical success was significantly associated with ‘complicated spines’ (P = 0.022) and the use of ultrasound assistance (P = 0.01), and the use of ultrasound was the only independent predictor of uncomplicated procedures (P = 0.007).

**Discussion:**

In adult patients with SMA both landmark‐based and ultrasound‐assisted techniques are safe and effective even in the long term. The use of assistance is associated with technical success and can predict uncomplicated procedures.

**Conclusion:**

Our results support the use of ultrasonography in order to improve the success and reduce the burden of nusinersen intrathecal administration.

## Introduction

Nusinersen (Spinraza, Biogen Inc., Boston, MA, USA), the first treatment approved for spinal muscular atrophy (SMA),^1^, ^2^ is an antisense oligonucleotide administered intrathecally through a lumbar puncture.^3^ This procedure can be challenging in some adult patients with neuromuscular scoliosis caused by muscle weakness, who are often treated with spinal instrumentation to stabilise the spine and prevent worsening deformities.^4^ In such patients, various devices (implantable catheters), surgical procedures (lumbar laminotomy) and imaging techniques (fluoroscopy‐, CT‐scans) have been used.^5^, ^6^, ^7^, ^8^, ^9^, ^10^, ^11^, ^12^ Ultrasound guidance and/or assistance has recently been introduced as a useful and successful tool in SMA patients with difficult spine.^13^, ^14^, ^15^, ^16^ Avoiding potential health risks (e.g. cancer) from ionising radiation and sedation, as repeated injections and exposures are needed, as per drug protocols, and allowing a more comfortable position for patients with joint contractures are several potential advantages of ultrasound compared with CT‐ or fluoroscopy‐guided techniques. Ultrasound guidance and/or assistance has been associated with a high success rate and a reduction in the number of needle insertion attempts required for successful anaesthesia,^17^ mainly in obstetric setting.^18^, ^19^, ^20^ A reduced risk of adverse events (AEs), such as post‐dural puncture headache (PDPH) and low back pain (LBP), and low patient satisfaction, often associated with multiple needle punctures, has also reported.^21^

The aim of this study was to evaluate the efficacy of the procedure, defined as the rate of successful intrathecal administration of nusinersen, number of attempts, procedure time and AEs in SMA adult patients using either ultrasound assistance or the landmark‐based technique.

## Materials and methods

### Study design

We retrospectively analysed 507 intrathecal procedures.

### Patients

Between April 2018 and August 2022, 51 adult patients with a genetically confirmed diagnosis of 5q SMA (13 SMA type 2 and 38 SMA type 3) referred to the NEuroMuscular Omnicentre (NeMO) of our institution were enrolled in this study. Written informed consent for lumbar puncture and intrathecal administration of the drug was obtained from all patients. The exclusion criteria were congenital coagulopathy, localised infections and increased intracranial pressure. All patients were instructed on the treatment procedures and related risks, explained as severe (spinal cord injury/epidural hematoma/ abdominal organ lesions/perforation) and mild (PDPH, LBP) AEs, and alternative approaches to access the intrathecal space (fluoroscopy) if the ultrasound and/or landmark‐based administrations were reported at any time as uncomfortable. Based on the history of previous back surgery and the Scoliosis Research Society criteria,^22^ the patients were assigned to one of two groups: Group 1 (uncomplicated spines, such as patients with no previous back surgery and mild or no scoliosis [Cobb's angle < 20°]) and Group 2 (complicated spines, such as patients with previous back surgery and moderate or severe scoliosis [Cobb's angle > 20°]). All procedures were performed by one of two anaesthetists with at least 5 years of experience in clinical anaesthesia and ultrasound‐assisted neuraxial techniques.

### Techniques

All procedures were performed with the patients placed in the most comfortable position (either seated or placed in a lateral decubitus position). After skin disinfection with 2% chlorhexidine in 70% alcohol, the patients were treated using either ultrasound assistance or a landmark‐based technique. Ultrasound assistance is mainly used for patients with complicated spines. Both techniques have been used in patients with uncomplicated spines.

The entry point of the needle was defined as follows:

#### Ultrasound‐assisted technique

**Figure 1:**
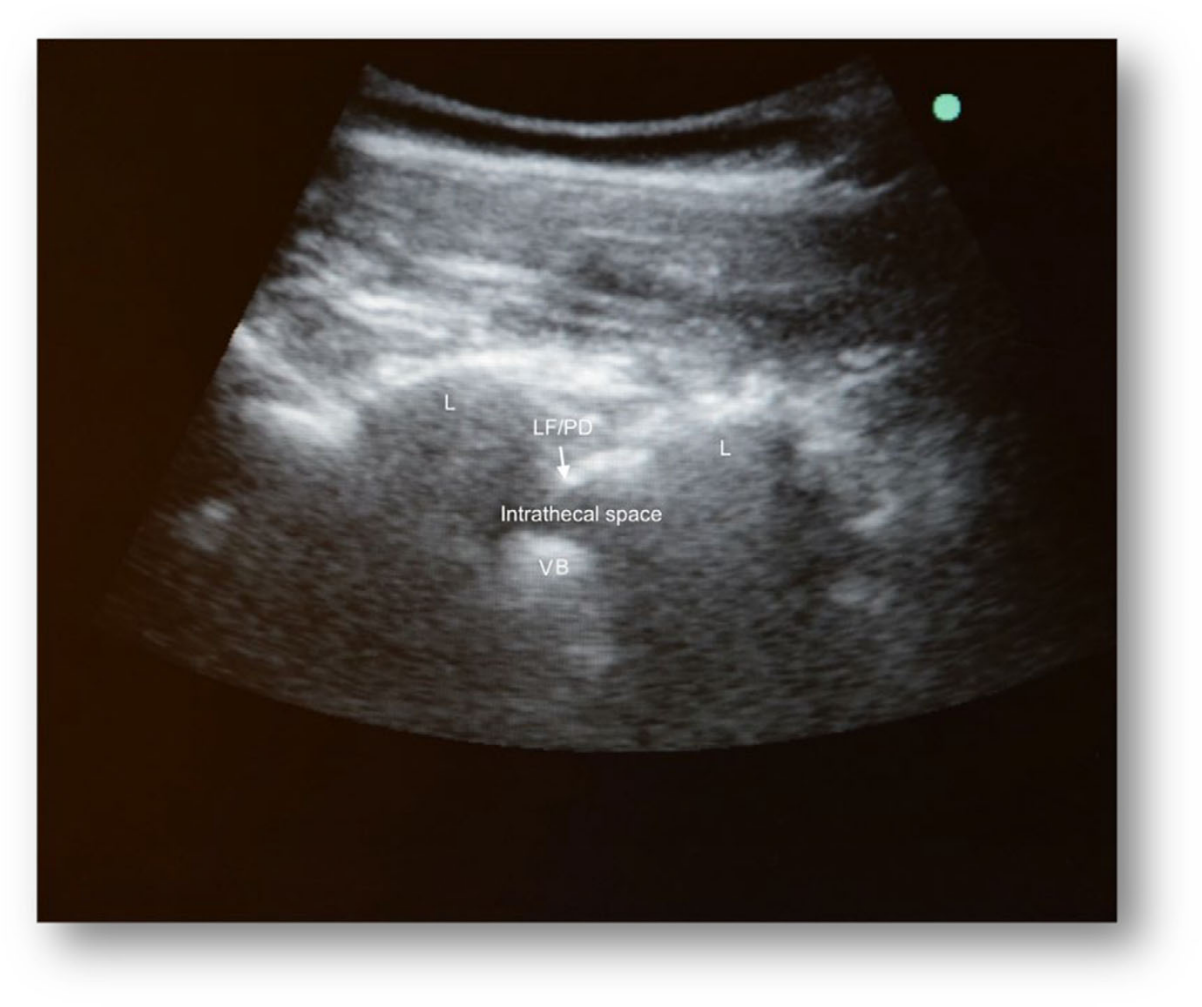
Paramedian sagittal oblique view of the lumbar spine. L, lumbar lamina; LF/PD, ligamentum flavum/posterior dura complex; VB, vertebral body.

**Figure 2:**
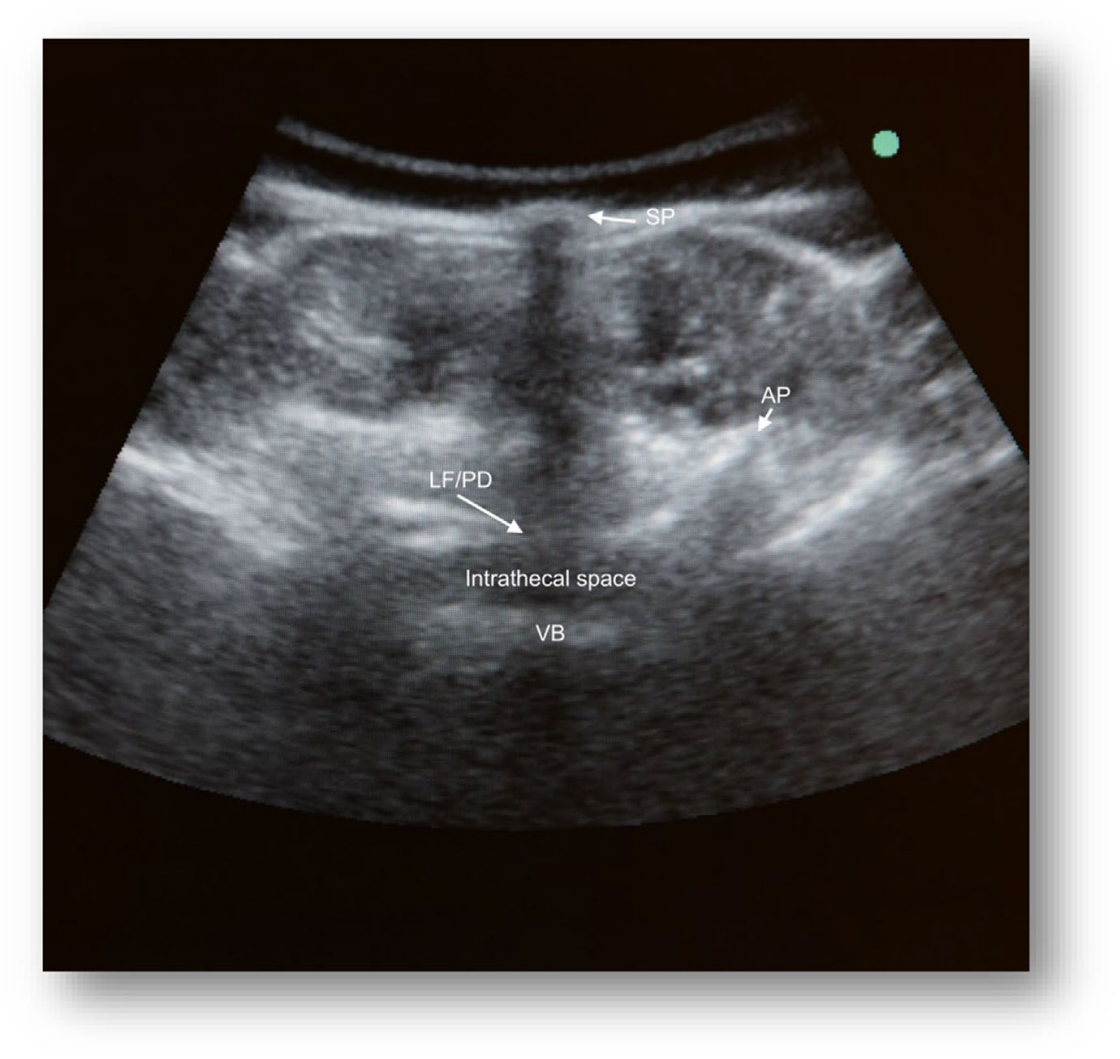
Transverse interlaminar view of the lumbar spine. AP, articular process; LF/PD, ligamentum flavum/posterior dura complex; SP, spinous process; VB, vertebral body.

A Sonosite M‐Turbo echograph and a convex array 2‐ to 5‐MegaHertz (MHz) transducer (Fujifilm Sonosite Europe, Amsterdam, Netherlands) were used. Using a sterile cover for the probe, starting at the sacrum and moving cephalad, the L3‐4 or L4‐5 intervertebral space was identified, and a left paramedian sagittal oblique view was obtained (Figure 1), which was rotated 90° into a transverse orientation, centred on the neuraxial midline, and moved in either the cephalad or caudal direction to obtain a transverse interlaminar view (Figure 2). The intersection between the two markings of the spinal midline and interlaminar space was identified as the spinal entry point of the needle. After local anaesthesia (LA) with lidocaine 2%, a 25‐gauge, non‐traumatic 90‐mm Whitacre spinal needle (BD Whitacre Needle; Becton Dickinson S.A., Madrid, Spain) or a traumatic 90‐mm Quincke spinal needle (BD Whitacre Needle, Becton Dickinson S.A.) was used to access the subarachnoid space; larger gauge needles were used to prevent their flexion or deviation from the intended trajectory through the tissues in scoliotic patients or in patients with previous back surgery. After confirmation of cerebrospinal fluid (CSF) flow, 5 mL of CSF was removed as per the drug protocol. Subsequently, nusinersen was administered intrathecally for 1–3 min.

#### Landmark‐based technique

Conventional palpation of the superior border of the iliac crest was performed, and the palpated intercristal line was assumed to intersect the spine at the L4 vertebral body or L4–L5 interspace. The skin was marked with horizontal and vertical lines at the L3–L4 or L2–L3 interspaces and was referred to as the entry point of the needle. The procedure was then completed in the same manner as for the ultrasound‐assisted technique.

### Injections and clinical evaluation

Injections occurred in three loading doses separated by 2 weeks, a fourth dose performed a month after the third, and indefinite maintenance doses every 4 months afterwards.^23^ After injection, patients were placed in the supine position for 2–3 h and then discharged. A phone survey was conducted at 24, 48 and 72 h to evaluate the development of severe or mild AEs.

### Variables collected

Outcomes were defined as follows:

- ‘Successful administration’, defined as a confirmation of the CSF flow through the spinal needle and subsequent administration of the total amount of the drug;
- ‘Failed administration’, defined as the inability to reach the intrathecal space;
- ‘Optimal procedure’, defined as a successful intrathecal administration with ≤4 attempts (number of needle insertions through the skin surface);
- ‘Suboptimal procedure’, defined as successful intrathecal administration requiring more than four attempts;
- ‘Procedure time’, defined as the amount of time measured in seconds from the start of the ultrasound imaging procedure or the palpation of the superior border of the iliac crest to the visualisation of CSF flow;
- ‘Adverse events’ were defined as mild AEs (PDPH, LBP) or severe AEs (spinal cord injury/epidural hematoma, abdominal organ lesions), according to the Society of Interventional Radiology (SIR) guidelines on AEs.^24^

### Statistical analysis

Continuous variables were reported as mean ± standard deviation or median (25th quartile–75th quartile) if not normally distributed. Normal distribution of a variable was assessed graphically using the Shapiro–Wilk test. Categorical variables are reported as cases (percentage). Differences in continuous variables were evaluated using the Student's *t*‐test or Wilcoxon rank‐sum test, as appropriate. Differences in categorical variables were evaluated using the chi‐squared test or Fisher's exact test, as appropriate.

We applied a generalised linear mixed model to evaluate the predictors of technical success and complications in the context of repeated procedures in the same patient. We used the patient as a random effect and included as fixed effects the factors known to be associated with these outcomes; body mass index (BMI), spine deformity, technique (ultrasound vs. landmark), type of needle and number of attempts were also included in the model for complications. Statistical significance was set at P < 0.05. All analyses were performed using R Statistical Computing, version 4.1, Vienna, Austria.

### Ethics approval

The study was approved by the Ethics Committee of Fondazione Policlinico Universitario A. Gemelli IRCCS (Protocol ID: 5384) and registered at ClinicalTrials. gov (NCT05644899).

## Results

### Demographic and clinical data

The demographic data are reported in Table 1. Fifty‐one consecutive patients were enrolled: 13 (25.5%) were affected by SMA type 2 and 38 (74.5%) by SMA type 3. The mean patient age was 43 ± 14 years, and the mean patient BMI was 22.4 ± 4.8 kg/m^2^, with no statistical differences between the two groups. Patients in Group 2 had a more severe form of SMA; two patients in Group 2 presented with a previous spinal fusion with instrumentation to correct spinal deformity.

**Table 1:** Demographic and clinical data.

	Total population (n = 51)	Group 1 (n = 26)	Group 2 (n = 25)	P value
SMA type 2 (n, %)	13 (25.5)	0 (0)	13 (52)	<0.001
SMA type 3 (n, %)	38 (74.5)	26 (100)	12 (48)	
Sex (male) (n, %)	26 (51)	10 (38.5)	16 (64)	0.068
Age (years)	43 ± 14	46 ± 15	40 ± 13	0.139
BMI (kg/m^2^)	22.4 ± 4.8	23.4 ± 3.5	21.3 ± 5.8	0.116
Treatments per patient (n)	10 ± 4	10 ± 4	10 ± 3	0.739

Data are shown as mean ± standard deviation or count (percentage) for the entire population. P‐value refers to Student's *t*‐test or chi‐squared test or Fisher's exact test, as appropriate.

BMI, Body Mass Index; Group 1, uncomplicated spines; Group 2, complicated spines; kg, kilograms; m, meter; n, number.

### Technical successes

Table 2 presents the results. Five hundred seven consecutive procedures were performed: 338 (66.7%) using ultrasound assistance and 169 (33.3%) using a landmark‐based technique. A significantly longer median time and a higher median number of attempts were required to perform the procedures in Group 2 than in Group 1 (217 [120–547] vs. 89 [60–120] seconds, P < 0.001 and 1 [1, 2] vs. 1 [1] attempt, P < 0.001, respectively). The overall optimal procedure rate was 90.3%, which was higher in uncomplicated spines than in complicated spines (96.1% vs. 84.7%, P < 0.001, respectively). We reported an overall success rate of 99.6%, with no statistical difference between the two groups (254 vs. 251, P = 0.249). A significantly higher number of procedures was performed using the Quincke needle than the Whitacre needle in Group 2 (99 vs. 8, P < 0.001). Two failed administrations were reported in Group 2: In one patient with severe scoliosis, the inability to reach the intrathecal space was reported; in the second patient, the procedure was interrupted due to the unintentional puncture of a bulky abdominal cyst. Of note, five procedures that failed with the landmark‐based technique were successfully performed on the same day after the pre‐procedural ultrasound scanning (conversion rate 2%).

**Table 2:** Technical aspects and adverse events.

	Total procedures (n = 507)	Group 1 (n = 254)	Group 2 (n = 253)	P value
Landmark (n, %)	169 (33.3)	114 (44.9)	55 (21.7)	<0.001
Ultrasound (n, %)	338 (66.7)	140 (55.1)	198 (78.3)	
Quincke 22 G (n, %)	107 (23.1)	8 (3.5)	99 (42)	<0.001
Whitacre 25 G (n, %)	357 (76.9)	220 (96.5)	137 (58)	
Time (s)^a^	120 (80, 300)	89 (60, 120)	217 (120, 547)	<0.001
Attempt number (n)^a^	1 (1, 2)	1 (1, 1)	1 (1, 2)	<0.001
Optimal procedure (n, %)^a^	419 (90.3)	219 (96.1)	200 (84.7)	<0.001
Successful administration (n, %)	505 (99.6)	254 (100)	251 (99.2)	0.249
Unsuccessful administration (n, %)	2 (0.4)	0 (0)	2 (0.8)	ns
Uncomplicated procedures (n, %)	455 (89.7)	227 (89.4)	228 (90.1)	0.781
PDPH (n, %)	27 (5.3)	16 (6.3)	11 (4.4)	0.328
Low back pain (n, %)	24 (4.7)	12 (4.7)	12 (4.7)	0.992
Conversion rate		5 (2%)	0 (0)	ns

Data are shown as median (25th–75th quartile) or count (percentage) for the entire population. P‐value refers to Wilcoxon rank‐sum test or chi‐squared or Fisher's exact test, as appropriate.

G, gauge; Group 1, uncomplicated spines; Group 2, complicated spines; n, number; PDPH, post dural puncture headache.

^a^ Of note, full data, including time and attempts, were available for 464 procedures. Optimal procedure is defined as successful drug administration with <4 attempts.

### Adverse events

A total of 455 procedures (89.7%) were performed uneventfully, with no statistically significant differences between the groups (Table 2). One (0.2%) severe AE was reported in Group 2: A bulky abdominal annexal cyst occupying the entire abdomen was touched, and a drainage of 3 mL of citrine liquid was obtained. The procedure was stopped and a CT scan of the abdomen was performed. The patient was observed for 48 h and discharged without any adverse sequelae. Fifty‐one mild AEs were reported, with no statistically significant differences between the two groups. Twenty‐seven episodes (5.3%) of PDPH, starting at 24 h, were reported and managed medically, with complete resolution within 72 h. Twenty‐four (4.7%) episodes of radicular or back pain were also reported, with only one patient developing both simultaneously; they were treated with non‐steroidal anti‐inflammatory drugs, and pain resolved within 1‐week post‐procedure.

### Factors associated with technical success and complications

The full results of the generalised linear mixed models are presented in Table 3. Successful administration was significantly associated with complicated spines (OR: 0.008 [0.01–0.07], P = 0.022) and the use of ultrasound assistance vs. landmark technique (OR: 5.2 [1.48–18.29], P = 0.01). The use of ultrasound was the only independent predictor of an uncomplicated procedure (OR: 0.36 [0.17–0.76], P = 0.007). Higher BMI and greater number of attempts showed a trend towards increased complications, although the difference was not significant.

**Table 3:** Factors associated with technical success and complications.

	Technical success		Complications	
	Estimated OR (95% CI)	P value	Estimated OR (95% CI)	P value
Intercept	2.96 (0.05–186.35)	0.607	0.04 (0–0.43)	0.007
BMI	1.11 (0.95–1.31)	0.178	1.08 (0.99–1.18)	0.081
Complicated spines	0.08 (0.01–0.7)	0.022	1.07 (0.45–2.51)	0.884
Ultrasound vs. landmark	5.2 (1.48–18.29)	0.01	0.36 (0.17–0.76)	0.007
25 G vs. 22 G needle	2.47 (0.93–6.57)	0.07	0.44 (0.17–1.13)	0.09
Number of attempts			1.06 (0.97–1.16)	0.206

Results of a generalised linear mixed model, using patients as random effect and body mass index (BMI), spine complexity (Group 2 vs. 1), technique, needle and number of attempts as fixed effects.

We report the estimated odd ratios (OR) with 95% confidence intervals (CI) and relative P‐values.

G, gauge.

## Discussion

Our retrospective analysis, which included a relatively large cohort of adult SMA patients who underwent a considerable number of procedures, showed the following:

- Both landmark‐based and ultrasound‐assisted techniques are safe and effective, even in the long term, after multiple procedures.
- The use of ultrasound allows for a reduced number of attempts to reach the intrathecal space, even in patients with complicated spines.
- Ultrasound assistance is an effective ‘rescue’ technique for failed landmark‐based procedures.
- The prevalence of AEs in our cohort was lower than that reported in the literature and ultrasound assistance was associated with fewer AEs.

The availability of new drugs with different routes of administration has been approved for the treatment of SMA,^25^, ^26^, ^27^, ^28^ prompting us to reflect on the safety and efficacy of intrathecal administration in the long term, especially for patients with complex spines. Similarly, reducing the burden of therapy with extensive evidence of efficacy^29^, ^30^ is a concern for physicians involved in the management of SMA. This retrospective analysis demonstrated that ultrasound‐assisted interlaminar lumbar puncture is safe and effective in SMA patients with different degrees of disability and age. Even though CT‐ and fluoroscopy‐guided procedures have been proposed for nusinersen administration, mainly in patients with difficult spines,^31^, ^32^, ^33^ a radiation‐free technique such as ultrasound could be desirable for patients requiring repetitive administrations. Recently, the ultrasound‐guided interlaminar or transforaminal lumbar approach has been proposed and presented as safe and effective, with a success rate between 50% and 100% in the adult SMA population.^13^, ^14^, ^15^ A possible advantage of ultrasound‐assisted vs. ultrasound‐guided technique could be the greater ease of execution, which is related to the very narrow acoustic window for the ultrasound beam at the lumbar level; advancing the needle under direct ultrasound guidance may not be easy. As a proof of this, we highlight our higher success rate than that of the other groups. Moreover, all procedures were performed under local anaesthesia, with no patients requiring sedation or general anaesthesia. Although reported as safe and effective in children treated with nusinersen for SMA type 2,^34^ sedation carries a number of risks and inconveniences and can prolong hospitalisation. In our experience, adults were safely treated under LA with a favourable cost‐effectiveness profile (discharge within 3 h from injection in all but one case). The overall procedure time we registered is shorter than that reported with other techniques,^35^ a relevant advantage with more favourable positioning throughout, as opposed to CT, which is often uncomfortable, stressful and painful for patients with joint contractures. A statistically longer procedure time was found in Group 2 than in Group 1. This result is not surprising and is in line with literature data: A significantly longer time required to perform a spinal injection by ultrasound imaging compared with palpation was registered in patients with difficult anatomical landmarks and abnormal spinal anatomy.^17^, ^36^ Although statistical significance was reached, we did not consider our results clinically significant when weighed against the benefits of the technique in this selected population of patients. All patients with scoliosis or previous surgery have a unique anatomy, and individual planning using ultrasound can be particularly helpful for spinal injections in these populations. The role of ultrasound as ‘rescue technique’ after a failed landmark‐based procedure is reported in the literature^36^ and confirmed in our study. Moreover, generalised linear mixed models showed that technical success was significantly associated with the use of ultrasound assistance. Our results seem to encourage the use of ultrasonography in patients with difficult spines, without concerns about prolonging the procedure time, as elsewhere reported.^37^

Only mild AEs, such as self‐limiting headache and back pain, were registered in both landmark‐based and ultrasound‐assisted techniques, regardless of needle size. The overall prevalence of headache found in our paper (5.3%) was lower than that reported in the literature. Konersman reported a procedure‐related headache occurring in 50% of adult subjects and 21% of 5–18 year‐old subjects^38^; a frequency between 2.2% and 60% was also described.^10^, ^13^, ^15^, ^35^, ^39^, ^40^ The lower incidence of headache, as well as LBP, can be related to two factors: the reduced number of attempts (therefore minimising the trauma to the spinal meninges and peridural membranes, which are abundant in nociceptors and other sensory receptors^41^); the use of ultrasound, defined in our statistical analysis as a single independent predictor associated with an uncomplicated procedure injection. We can suppose that the ultrasound allowed a targeted procedure, thereby avoiding contact and possible injury to sensitive nearby structures. The same conclusion can be applied to our patient with a bulky abdominal annexal cyst occupying the entire abdomen. In our opinion, the use of ultrasound allowed multiple procedures to be performed without other major complications.

This retrospective study has several limitations. First, it was a single‐centre study. Some recommendations regarding ultrasound assistance may not be generalisable to all centres. Local expertise, resource limitations and availability of physicians with expertise in neuraxial procedures can impact both easy and challenging LPs.^42^ Second, compared with other studies,^11^, ^13^, ^14^ a smaller number of patients with growing rods were referred to our center and enrolled in this study. Further studies enrolling a larger number of patients with previous back surgery are needed to confirm the ultrasound‐assistance as useful in patients in whom the lack of spinous processes as anatomical landmarks can make the procedure very challenging.

## Conclusion

In adult patients with SMA, both landmark‐based and ultrasound‐assisted techniques are safe and effective, even in the long term. The use of assistance is associated with technical success in patients with complicated spines and predicts uncomplicated procedures. These results, as well as our previous report,^16^ seem to encourage the use of ultrasonography, mainly in patients with difficult spines, in order to improve the success of the procedure and reduce the burden of intrathecal administration.

## Author contributions

**Bruno Antonio Zanfini:** Conceptualization (lead); data curation (equal); investigation (equal); writing – original draft (equal); writing – review and editing (equal). **Agata Katia Patanella:** Conceptualization (equal); investigation (equal); resources (equal); writing – review and editing (equal). **Francesco Vassalli:** Data curation (equal); formal analysis (equal); software (equal). **Stefano Catarci:** Investigation (equal); validation (equal). **Marika Pane:** Writing – review and editing (equal). **Luciano Frassanito:** Writing – review and editing (equal). **Matteo Biancone:** Data curation (equal); resources (equal). **Mariangela Di Muro:** Data curation (equal); resources (equal). **Chiara Bravetti:** Writing – review and editing (equal). **Eugenio Maria Mercuri:** Supervision (equal). **Mario Sabatelli:** Supervision (equal). **Gaetano Draisci:** Supervision (equal).

## Conflict of interest

Bruno Antonio Zanfini has served as a paid consultant for BIOGEN S.R.L. outside the submitted work; Agata Katia Patanella has served as a paid consultant for BIOGEN S.R.L. outside the submitted work; Marika Pane has served as a paid consultant for BIOGEN S.R.L. and Novartis outside the submitted work; Eugenio Mercuri has served as a paid consultant for BIOGEN S.R.L., Novartis and Roche outside the submitted work; Mario Sabatelli has served as a paid consultant for BIOGEN S.R.L. outside the submitted work; the remaining authors have no conflicts of interest to disclose.

## References

[1] Commissioner of the FDA approves first drug for spinal muscular atrophy. 2019. Available from: http://www.fda.gov/news‐events/press‐announcements/fda‐approves‐first‐drug‐spinal‐muscular‐atrophy.

[2] Summary of the European public assessment report (EPAR) Spinraza. Available from: https://www.ema.europa.eu/en/medicines/human/EPAR/spinraza#product‐information‐section.

[3] Geary RS , Yu RZ , Levin AA . Pharmacokinetics of phosphonothioate antisense oligodeoxynucleotides. Curr Opin Investig Drugs 2001; 2: 562–573. 11566019

[4] Lunn MR , Wang CH . Spinal muscular atrophy. Lancet 2008; 371: 2120–2133. 18572081 10.1016/S0140-6736(08)60921-6

[5] Strauss KA , Carson VJ , Brigatti KW , Young M , Robinson DL , Hendrickson C , *et al*. Preliminary safety and tolerability of a novel subcutaneous intrathecal catheter system for repeated outpatient dosing of nusinersen to children and adults with spinal muscular atrophy. J Pediatr Orthop 2018; 38: e610–e617. 30134351 10.1097/BPO.0000000000001247 PMC6211782

[6] Iannaccone ST , Paul D , Castro D , Weprin B , Swift D . Delivery of Nusinersen through an Ommaya reservoir in spinal muscular atrophy. J Clin Neuromuscul Dis 2021; 22: 129–134. 33595996 10.1097/CND.0000000000000333

[7] Papaliagkas V , Foroglou N , Toulios P , Moschou M , Gavriilaki M , Notas K , *et al*. Intrathecal Administration of Nusinersen Using the Ommaya reservoir in an adult with 5q‐related spinal muscular atrophy type 1 and severe spinal deformity. Case Rep Neurol 2021; 13: 710–715. 34950009 10.1159/000519831 PMC8647073

[8] Ko D , Blatt D , Karam C , Gupta K , Raslan AM . Lumbar laminotomy for the intrathecal administration of nusinersen for spinal muscular atrophy: technical note and outcomes. J Neurosurg Spine 2019; 31: 217–222. 31003222 10.3171/2019.2.SPINE181366

[9] Bortolani S , Stura G , Ventilii G , Vercelli L , Rolle E , Ricci F , *et al*. Intrathecal administration of nusinersen in adult and adolescent patients with spinal muscular atrophy and scoliosis: transforaminal versus conventional approach. Neuromuscul Disord 2019; 29: 742–746. 31604650 10.1016/j.nmd.2019.08.007

[10] Özütemiz C , Karachunski P , Nascene DR . Nusinersen injections in adults and children with spinal muscular atrophy: a single‐center experience. Diagn Interv Radiol 2020; 26: 596–602. 32436843 10.5152/dir.2020.19607 PMC7664748

[11] Weaver JJ , Hallam DK , Chick JFB , Vaidya S , Shin DS , Natarajan N , *et al*. Transforaminal intrathecal delivery of nusinersen for older children and adults with spinal muscular atrophy and complex spinal anatomy: an analysis of 200 consecutive injections. J Neurointerv Surg 2021; 13: 75–78. 32471828 10.1136/neurintsurg-2020-016058

[12] Johannsen J , Weiss D , Schlenker F , Groth M , Denecke J . Intrathecal Administration of Nusinersen in pediatric SMA patients with and without spine deformities: experiences and challenges over 3 years in a single center. Neuropediatrics 2021; 52: 179–185. 33276405 10.1055/s-0040-1718916

[13] Veiga‐Canuto D , Cifrián‐Pérez M , Pitarch‐Castellano I , Vázquez‐ Costa JF , Aparici F . Ultrasound‐guided lumbar puncture for nusinersen administration in spinal muscular atrophy patients. Eur J Neurol 2021; 28: 676–680. 33051940 10.1111/ene.14586

[14] Snoj Ž , Salapura V . Ultrasound‐guided transforaminal approach for nusinersen delivery in adult spinal muscle atrophy patients with challenging access. Muscle Nerve 2022; 65: 585–589. 35147227 10.1002/mus.27518

[15] Zhang J , Cui X , Chen S , Dai Y , Huang Y , Zhang S . Ultrasound‐guided nusinersen administration for spinal muscular atrophy patients with severe scoliosis: an observational study. Orphanet J Rare Dis 2021; 16: 274. 34120632 10.1186/s13023-021-01903-4 PMC8201867

[16] Zanfini BA , Catarci S , Patanella AK , Pane M , Frassanito L , Filipponi E , *et al*. Ultrasound assisted lumbar intrathecal administration of nusinersen in adult patients with spinal muscular atrophy: a case series. Muscle Nerve 2021; 64: 594–599. 34396547 10.1002/mus.27400

[17] Chin KJ , Perlas A , Chan V , Brown‐Shreves D , Koshkin A , Vaishnav V . Ultrasound imaging facilitates spinal anesthesia in adults with difficult surface anatomic landmarks. Anesthesiology 2011; 115: 94–101. 21572316 10.1097/ALN.0b013e31821a8ad4

[18] Vallejo MC , Phelps AL , Singh S , Orebaugh SL , Sah N . Ultrasound decreases the failed labor epidural rate in resident trainees. Int J Obstet Anesth 2010; 19: 373–378. 20696564 10.1016/j.ijoa.2010.04.002

[19] Tawfik MM , Atallah MM , Elkharboutly WS , Allakkany NS , Abdelkhalek M . Does preprocedural ultrasound increase the first‐pass success rate of epidural catheterization before cesarean delivery? A randomized controlled trial. Anesth Analg 2017; 124: 851–856. 27183373 10.1213/ANE.0000000000001325

[20] Kallidaikurichi Srinivasan K , Iohom G , Loughnane F , Lee PJ . Conventional landmark‐guided midline versus preprocedure ultrasound‐guided paramedian techniques in spinal anesthesia. Anesth Analg 2015; 121: 1089–1096. 26270115 10.1213/ANE.0000000000000911

[21] Horlocker TT , McGregor DG , Matsushige DK , Schroeder DR , Besse JA . A retrospective review of 4767 consecutive spinal anesthetics: central nervous system complications. Perioperative outcomes group. Anesth Analg 1997; 84: 578–584. 9052305 10.1097/00000539-199703000-00021

[22] Smith JS , Shaffrey CI , Kuntz C 4th , Mummaneni PV . Classification systems for adolescent and adult scoliosis. Neurosurgery 2008; 63: 16–24. 18812919 10.1227/01.NEU.0000320447.61835.EA

[23] Spinraza‐prescribing‐information.pdf. Available from: https://www.spinraza.com/content/dam/commercial/specialty/spinraza/caregiver/en_us/pdf/spinraza‐prescribinginformation.pdf.

[24] Khalilzadeh O , Baerlocher MO , Shyn PB , Connolly BL , Devane AM , Morris CS , *et al*. Proposal of a new adverse event classification by the Society of Interventional Radiology Standards of practice committee. J Vasc Interv Radiol 2017; 28: 1432–1437. 28757285 10.1016/j.jvir.2017.06.019

[25] Michelson D , Ciafaloni E , Ashwal S , Lewis E , Narayanaswami P , Oskoui M , *et al*. Evidence in focus: Nusinersen use in spinal muscular atrophy: report of the guideline development, dissemination, and implementation Subcommittee of the American Academy of neurology. Neurology 2018; 91: 923–933. 30315070 10.1212/WNL.0000000000006502

[26] Mercuri E , Darras BT , Chiriboga CA , Day JW , Campbell C , Connolly AM , *et al*. Nusinersen versus sham control in later‐onset spinal muscular atrophy. N Engl J Med 2018; 378: 625–635. 29443664 10.1056/NEJMoa1710504

[27] Baranello G , Darras BT , Day JW , Deconinck N , Klein A , Masson R , *et al*. Risdiplam in type 1 spinal muscular atrophy. N Engl J Med 2021; 384: 915–923. 33626251 10.1056/NEJMoa2009965

[28] Stevens D , Claborn MK , Gildon BL , Kessler TL , Walker C . Onasemnogene Abeparvovec‐xioi: gene therapy for spinal muscular atrophy. Ann Pharmacother 2020; 54: 1001–1009. 32204605 10.1177/1060028020914274

[29] Coratti G , Cutrona C , Pera MC , Bovis F , Ponzano M , Chieppa F , *et al*. Motor function in type 2 and 3 SMA patients treated with Nusinersen: a critical review and meta‐analysis. Orphanet J Rare Dis 2021; 16: 430. 34645478 10.1186/s13023-021-02065-z PMC8515709

[30] Pane M , Coratti G , Pera MC , Sansone VA , Messina S , d'Amico A , *et al*. Nusinersen efficacy data for 24‐month in type 2 and 3 spinal muscular atrophy. Ann Clin Transl Neurol 2022; 9: 404–409. 35166467 10.1002/acn3.51514 PMC8935309

[31] Nascene DR , Ozutemiz C , Estby H , McKinney AM , Rykken JB . Transforaminal lumbar puncture: an alternative technique in patients with challenging access. Am J Neuroradiol 2018; 39: 986–991. 29567652 10.3174/ajnr.A5596 PMC7410647

[32] Ghai B , Bansal D , Kay JP , Vadaje KS , Wig J . Transforaminal versus parasagittal interlaminar epidural steroid injection in low back pain with radicular pain: a randomized, double‐blind, active‐control trial. Pain Physician 2014; 17: 277–290. 25054387

[33] Oldenburg D , Guberina N , Stolte B , Kizina K , Stenzel E , Radbruch A , *et al*. Radiation exposure of image‐guided intrathecal administration of nusinersen to adult patients with spinal muscular atrophy. Neuroradiology 2019; 61: 565–574. 30868184 10.1007/s00234-019-02189-x

[34] Bielsky AR , Fuhr PG , Parsons JA , Yaster M . A retrospective cohort study of children with spinal muscular atrophy type 2 receiving anesthesia for intrathecal administration of nusinersen. Paediatr Anaesth 2018; 28: 1105–1108. 30284761 10.1111/pan.13500

[35] Cordts I , Lingor P , Friedrich B , Pernpeintner V , Zimmer C , Deschauer M , *et al*. Intrathecal nusinersen administration in adult spinal muscular atrophy patients with complex spinal anatomy. Ther Adv Neurol Disord 2020; 13: 1756286419887616. 32010224 10.1177/1756286419887616 PMC6974755

[36] Park SK , Bae J , Yoo S , Kim WH , Lim YJ , Bahk JH , *et al*. Ultrasound‐assisted versus landmark‐guided spinal anesthesia in patients with abnormal spinal anatomy: a randomized controlled trial. Anesth Analg 2020; 130: 787–795. 31880632 10.1213/ANE.0000000000004600

[37] Gottlieb M , Holladay D , Peksa GD . Ultrasound‐assisted lumbar punctures: a systematic review and meta‐analysis. Acad Emerg Med 2019; 26: 85–96. 30129102 10.1111/acem.13558

[38] Konersman CG , Ewing E , Yaszay B , Naheedy J , Murphy S , Skalsky A . Nusinersen treatment of older children and adults with spinal muscular atrophy. Neuromuscul Disord 2021; 31: 183–193. 33608138 10.1016/j.nmd.2020.12.006

[39] Hagenacker T , Wurster CD , Günther R , Schreiber‐Katz O , Osmanovic A , Petri S , *et al*. Nusinersen in adults with 5q spinal muscular atrophy: a non‐interventional, multicentre, observational cohort study. Lancet Neurol 2020; 19: 317–325. 32199097 10.1016/S1474-4422(20)30037-5

[40] Salapura V , Snoj Z , Leonardis L , Koritnik B , Kostadinova V . Cone‐beam computed tomography guided nusinersen administrations in adult spinal muscular atrophy patients with challenging access: a single‐ center experience. Radiol Oncol 2022; 56: 319–325. 35962954 10.2478/raon-2022-0033 PMC9400441

[41] Sehgal I , Das JM . Anatomy, back, spinal meninges. In: StatPearls [Internet]. Treasure Island, FL: StatPearls Publishing; 2022. 31613535

[42] Moshe‐Lilie O , Visser A , Chahin N , Ragole T , Dimitrova D , Karam C . Nusinersen in adult patients with spinal muscular atrophy: observations from a single center. Neurology 2020; 95: e413–e416. 32665408 10.1212/WNL.0000000000009914

